# Pancreatic Cancer Patient-Derived Organoid Platforms: A Clinical Tool to Study Cell- and Non-Cell-Autonomous Mechanisms of Treatment Response

**DOI:** 10.3389/fmed.2021.793144

**Published:** 2021-12-23

**Authors:** Geny Piro, Antonio Agostini, Alberto Larghi, Giuseppe Quero, Carmine Carbone, Annachiara Esposito, Gianenrico Rizzatti, Fabia Attili, Sergio Alfieri, Guido Costamagna, Giampaolo Tortora

**Affiliations:** ^1^Department of Medical and Surgical Sciences, Medical Oncology, Fondazione Policlinico Universitario Agostino Gemelli IRCCS, Roma, Italy; ^2^Digestive Endoscopy Unit, Fondazione Policlinico Universitario A. Gemelli IRCCS, Rome, Italy; ^3^CERTT, Center for Endoscopic Research Therapeutics and Training, Catholic University, Rome, Italy; ^4^Department of Surgery, Fondazione Policlinico Universitario Agostino Gemelli IRCCS, Rome, Italy; ^5^Department of Translational Medicine, Medical Oncology, Catholic University of the Sacred Heart, Rome, Italy; ^6^Comprehensive Cancer Center, Fondazione Policlinico Universitario Agostino Gemelli IRCCS, Rome, Italy

**Keywords:** patient derived organoid, pancreatic cancer, precision medicine, EUS-guided sampling, tumor-stroma heterogeneity

## Abstract

For many years, cell lines and animal models have been essential to improve our understanding of the basis of cell metabolism, signaling, and genetics. They also provided an essential boost to cancer drug discovery. Nevertheless, these model systems failed to reproduce the tumor heterogeneity and the complex biological interactions between cancer cells and human hosts, making a high priority search for alternative methods that are able to export results from model systems to humans, which has become a major bottleneck in the drug development. The emergent human *in vitro* 3D cell culture technologies have attracted widespread attention because they seem to have the potential to overcome these limitations. Organoids are unique 3D culture models with the ability to self-organize in contained structures. Their versatility has offered an exceptional window of opportunity to approach human cancers. Pancreatic cancers (PCs) patient-derived-organoids (PDOs) preserve histological, genomic, and molecular features of neoplasms they originate from and therefore retain their heterogeneity. Patient-derived organoids can be established with a high success rate from minimal tissue core specimens acquired with endoscopic-ultrasound-guided techniques and assembled into platforms, representing tens to hundreds of cancers each conserving specific features, expanding the types of patient samples that can be propagated and analyzed in the laboratory. Because of their nature, PDO platforms are multipurpose systems that can be easily adapted in co-culture settings to perform a wide spectrum of studies, ranging from drug discovery to immune response evaluation to tumor-stroma interaction. This possibility to increase the complexity of organoids creating a hybrid culture with non-epithelial cells increases the interest in organoid-based platforms giving a pragmatic way to deeply study biological interactions *in vitro*. In this view, implementing organoid models in co-clinical trials to compare drug responses may represent the next step toward even more personalized medicine. In the present review, we discuss how PDO platforms are shaping modern-day oncology aiding to unravel the most complex aspects of PC.

## Introduction

Pancreatic cancer (PC) is a highly aggressive disease with a very dismal prognosis and a generalized poor treatment response (1). Despite extensive research efforts, response rates remain poor, with a 5-year overall survival rate of <5% for locally advanced unresectable disease (2, 3).

High-throughput analyses have uncovered a clear diversity in pancreatic tumors at multiple molecular levels with a high degree of mutational and expression landscape heterogeneity (4–6). Several cancer subtyping with specific RNA signature has been proposed (4, 7, 8).

Stroma and immune cells are known to contribute to disease pathogenesis and response to therapy (9). Tumors with a high activated stroma index, low collagen abundance, and a dense αSMA positive cell infiltration had a worse prognosis (10). Moreover, besides cancer cell heterogeneity, it is now widely accepted the existence of more than one specific stroma subtype. Indeed, global transcriptomic profiling has revealed the presence of a “normal” and an “activated” stroma subtype with different abilities to influence patient prognosis (7). Cancer-associated fibroblasts (CAFs) drive crucial variations of the tumor microenvironment (TME) and interact with the immune compartment (11, 12). Recently, the single-cell analysis highlighted two functionally distinct pancreatic fibroblast lineages with mutually exclusive tumor suppressive or permissive properties (13).

For many years, traditional cell lines, xenograft, or genetically engineered mouse models (GEMMs) have significantly contributed to increasing our knowledge about the mechanism underlining PC pathology and progression. However, at present, the interest in the non-tumoral determinant of disease has greatly increased, and more reliable models are needed. Standard cancer models failed to fully replicate human disease as they lack non-tumor microenvironment elements. Patient-derived organoids (PDOs) seem to respond to this need. Organoids are unique 3D cultures that faithfully enclose the main characteristics of parental tissue they come from. Although the successful establishment of tumor organoids is mostly dependent on sample's basic characteristics such as quality, volume, and tissue origin, the derivation rate of organoids is largely higher than that of 2D cancer cell line stabilization from the same fresh tumor tissue (14).

Isolation and culture methods *in vitro* to propagate patient-derived PC organoids were successfully established by Tuveson and Clevers groups by dramatically changing the research models paradigm (15).

## The Need for Biologically Accurate Cancer Models for PC Study

For years, established cell lines from primary tumors were the main cancer models. They are easy and cheap to maintain and can be used for a wide range of genetic, biochemical, and pharmacological evaluations. Additionally, they were instrumental in the primordial phase of cancer drug development. In recent years, cell lines from primary tumors were also massively utilized in high throughput screening of antineoplastic agents. However, traditional 2D cell cultures failed to recapitulate the complex biological interactions of *in vivo* models. Indeed, 2D cultures do not reproduce key signaling pathways, such as cell-cell and cell-matrix interactions, which contribute to essential cellular functions in proliferation, differentiation, survival, and response to treatment (16, 17). In addition, cell lines generally originate from single cancer clones, thus lacking to represent the full heterogeneity of parental tumors that is one of the main causes of therapy response failure.

Conversely, *ex vivo* culture of organ slices allows some limited investigation on stroma cells, although long-term cell viability is poor and they cannot be expanded, stored, genetically manipulated, or used for immune cell migration studies (18).

Patient-derived xenografts (PDXs) obtained by engrafting neoplastic tissue into immuno-deficient mice or human immune-reconstituted mice can partially overcome these problems, recreating most of the key cell-to-cell and cell-to-matrix interactions. However, these models are both resource and time-consuming, and the corresponding results fail to fall within a clinically meaningful timeframe to guide the decision-making process. High-throughput screening of anticancer agents using xenografts is inconceivable, underlying the need for better, easily manageable, and adaptable cancer models, which develop as cell lines but preserve the same patient specificity as PDX.

To fulfill this need, PDOs were established from many types of cancers becoming a promising tool for personalized oncology nowadays. These models can fully reproduce the spectrum of tumor development, can be easily maintained, manipulated, and assembled in platforms representing tens to hundreds of patient-specific tumors for massive analysis of treatment response, i.e., pharmacotyping, biomarker discovery, and study of tumor-related pathways.

## PC PDOS Retain Parental Cancer Features

Organoids are 3D *in vitro* cellular structures derived from tissue-specific stem cells, with the capacity to self-organize in structures resembling the tissue of origin. In comparison to other culturing techniques, organoids are relatively simple to maintain and expand, thus offering various alternative modalities to assess treatment response to anti-tumoral agents.

Several studies showed that PDOs faithfully represent the main genomic and transcriptomic features of the tissue of origin and can be used as patient-specific avatars of disease (19–21). Importantly, they should be considered as immortalized avatars because they are stable over many passages, can be cryopreserved, and exchangeable between different laboratory settings, favoring collaborations among institutions across the world.

The organoid technology started with the exploitation of the proliferation properties of stem cells present in human tissues. Sasai group pioneered this technology creating pluripotent stem cell (PSC)-based organoids, which resembled the cytoarchitecture of specific regions of the central nervous system (22, 23). The subsequent work by Clevers group paved the way for the generation of PDOs. They were the first in 2009 to establish organoids from mouse colon crypts, exploiting the LRG5+ stem cells present at the base of the crypts. They discovered that modulating these cells with the combination of R-spondin-1 (Lgr5 ligand and Wnt signaling trigger), epidermal growth factor (EGF), and bone morphogenetic protein (BMP) inhibitor noggin, 3D epithelial structures embedded in the basement membrane matrix structure (organoids) could be created (24). They were then able to create intestinal organoids from patient-derived normal and colorectal cancer tissue adding Wnt, the transforming growth factor-β (TGF-β) inhibitor A83-01, and the p38 inhibitor SB202190 (25). Subsequently, PDOs were established from several types of cancer, such as pancreatic (15, 26), prostate (27, 28), gastric (29), and other cancers (20, 21, 30).

Nowadays, the establishment of organoids is generally easy, as many standardized protocols and commercial kits are available. Following tumor resection or biopsy, tissue is enzymatically and/or mechanically digested. The digested sample is then filtered with cell strainers and embedded into a protein-rich matrix such as Matrigel. Culture media usually differs to accommodate the growth conditions of the disparate types of cancer cells, but the modulators described above are all essential for organoid growth and expansions. Moreover, Y-27362, a Rho-associated coiled-coil protein kinase (ROCK) inhibitor, is generally added as it is essential to promote the growth of organoids and prevent anoikis (31).

In 2015, Boj et al. (15) established pancreatic organoid models from normal and neoplastic murine and human pancreatic resected tumors and biopsies for the first time. Such pancreatic organoids survived cryopreservation, displayed ductal features, and are representative of disease stage, tumor tissue organization, and pathology. When orthotopically transplanted in mice, organoids reproduced early-grade pre-invasive pancreatic intraepithelial neoplasms (PanIn) that progress to locally invasive and then metastatic carcinomas. Indeed, while transplanted 2D cell lines rapidly evolve in advanced disease with often null stroma response, organoid pancreatic transplants exhibit the ability to recapitulate both the discrete characteristic stages of normal *in vivo* disease progression and a prominent stromal response with low vascular density.

In the same year, Huang et al. (32) further demonstrated that it was possible to generate PDO from freshly resected pancreatic ductal adenocarcinoma (PDAC) maintaining differentiation status, histoarchitecture, heterogeneity, and patient-specific physiologic characteristic, including hypoxia, oxygen consumption, and epigenetic markers.

As unresectable cases represent about 85% of patients with PC (33), it has been fruitfully explored the derivation of organoids from the limited tissue amount of endoscopic biopsies with fine needle aspiration (FNA). To avoid material loss during digestion, which is the major limitation of this protocol, Boj et al. (15) successfully optimized experimental conditions removing dissociation steps prior to Matrigel suspension and granting efficient organoid generation.

Moreover, it has been recently reported a detailed and reliable protocol for generating orthotopic isograft mouse models using PC organoids in order to globally standardize the establishment of *in vivo* organoid-based tools for PDAC progression study (34).

Classical anchorage-independent spheroid culture derived from established human 2D cell lines or cells propagated in xenograft models are subjected to selective pressure from adherent culture technic or *in vivo* grow conditions and fails to replicate the original structure and polarity. Contrariwise, primary PDOs freshly derived from samples of patients grow much slower and start to self-organize mimicking tumor tissue cytoarchitecture with a lumen and apicobasal polarity and major pathophysiological features, cellular heterogeneity, mutational profile, stemness, and tumor niche (35).

Studies on histopathological and immunological evolution of genetically defined pancreatic organoid-derived isografts revealed a tight resembling of human natural disease development along each progression step from PanIN-like lesion to invasive and metastatic carcinoma confirming organoid *in vivo* development as a suitable model. Characterization of tumor cellular components at different times post-transplantation together with dynamic changes of the immunological context during progression showed that, as in patients, pre-invasive lesions were heavily infiltrated by granulocytes, macrophages, and regulatory T cells (Treg) cells that endured through progression, whereas helper CD^4+^ T and cytotoxic CD^8+^ T cells were excluded as the tumor progresses and degree of myeloid-derived suppressor cells (g-MDSC) and M2 macrophages increased (36).

Accordingly, scRNA-seq of PDAC organoids from human primary tumors and metastatic specimens showed an extensive agreement of subtype assignments with tumors of origin indicating that differences between PDAC patients are preserved in such cultures. Moreover, PDOs were characterized by cell state heterogeneity, and a conserved differentiation hierarchy also presented in primary PDACs (37).

## Sampling Issue: PC Endoscopic Ultrasound (EUS) VS. Surgery

A critical issue influencing the success rate of PDO establishment is the availability of an appropriate tissue sample of good quality.

**Figure 1:**
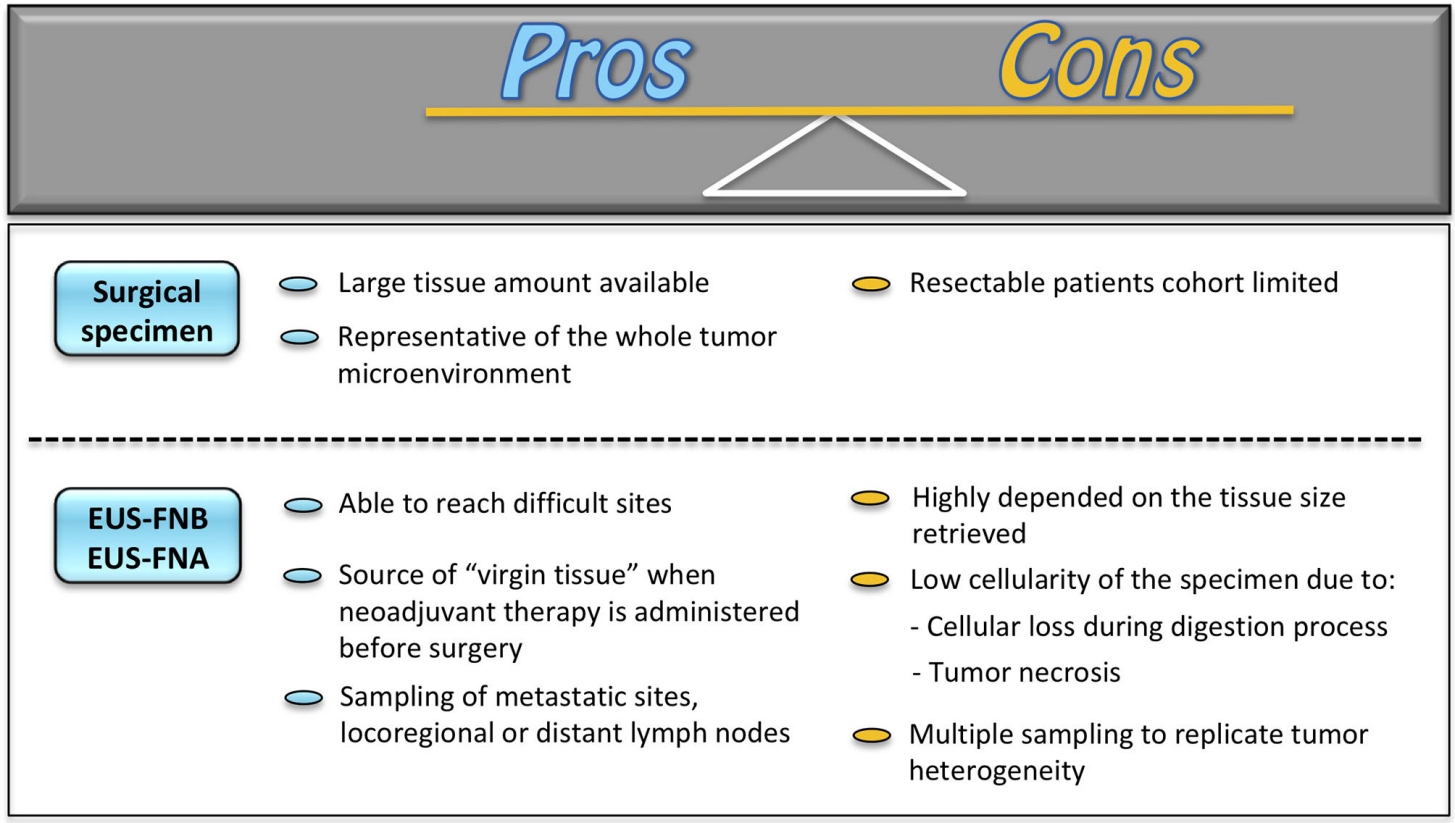
Surgery vs. EUS technics for PDO platform generation. EUS-FNA, EUS-guided fine needle aspiration; EUS-FNB, EUS-guided fine needle biopsy.

Patient-derived organoids have been firstly generated from surgically resected specimens. This approach provides a large amount of tissue for PDO culture and growth, preserving at the same time the quality of the tissue itself. Indeed, given the tumor environment heterogeneity, PDO creation from resected tumors has the advantage of appropriately recapitulating tumor phenotype and histoarchitecture while retaining molecular characteristics of primary malignancies (38). On the counterpart, this methodology presents undeniable drawbacks. Specifically, PDO creation from surgical specimens limits tumor knowledge only to resectable tumors. This would imply the exclusion of unresectable tumors, chemo-resistant lesions, and recurrent malignancies, where surgery is not an option. To overcome these limitations and to guarantee a more comprehensive analysis of tumor biology, tissue acquisition for PDO creation under EUS guidance has been recently introduced (Figure 1). The EUS-guided technique is able to reach sites otherwise difficult to be reached, such as the mediastinum, the retroperitoneum, the pelvis, and any lesion adjacent to the upper and lower (up to the sigma) gastrointestinal tract and to acquire tissue samples under real-time ultrasound control (39, 40). Moreover, EUS sampling can be a source of “virgin tissue” when neoadjuvant therapy is administered before surgery, an approach increasingly used for many tumors, and for locally advanced or borderline resectable lesions. In these latter situations, the capability of PDO drugs testing would have a major impact on the development of alternative therapeutic agents, i.e., pharmacotyping. Additionally, the EUS-guided technique may allow sampling not only of the primary tumor but also of metastatic sites, such as those in the left liver lobe, and locoregional or distant lymph nodes. This has the potential to recapitulate the full spectrum of tumorigenesis (15), which may represent an opportunity for probing such PDOs at the genomic level, as demonstrated for PDAC (41).

Despite these premises, a validation of EUS-guided sampling as a valuable alternative to surgery is still necessary. As a matter of fact, PDO creation from the biopsy is highly dependent on the tissue size retrieved, on the biopsy site, and on the consequent PDO expansion rate (42). Indeed, the presence of tumor necrosis and stromal cells in biopsies may reflect a low tumor cell percentage. Moreover, the digestion process as part of PDO creation may bring to a significant loss of cellular material leading to a further reduction of tumor cell isolation. This inevitably reflects in less accurate detection of somatic mutations and copy number aberrations, as compared to PDOs created with tissue cultures from resected specimens (43). Furthermore, the current evidence on the role of EUS tissue sampling is still based on pilot studies with a paucity of patients involved. In addition, “matching” studies of EUS samples with surgical specimen-derived organoids are still limited. It would be necessary to understand if the EUS-guided technique may be comparable to surgical tissue retrieval and, thus, be representative of the whole tumor biology.

These findings brought many researchers to specifically focus on the potential technical improvements in the EUS approach. Current techniques of EUS tissue sampling comprise EUS-guided FNA (EUS-FNA) and EUS-guided fine needle biopsy (EUS-FNB) (44). Samples are smeared on glass slides and then fixed in alcohol and sent to the cytology lab. Part of the obtained material can also be placed in a solution for cell-block evaluation (45). For EUS-FNB, specifically designed 22-gauge or 20-gauge needles are utilized and the collected material is directly placed in formalin and processed as a biopsy sample. For both methods, rapid on-site evaluation (ROSE) by a cytopathology/cytotechnician may be performed on cytological smears collected by FNA or on tissue samples smeared on a glass slide using the touch imprint cytology technique. This would allow determining the adequacy of the collected specimens and acquisition of further samples for research purposes, i.e., organoid.

The first report of PDOs from EUS-FNA of PDAC comes from Boj et al. (15) who were able to generate human FNA biopsy organoids from two specimens. Most of the subsequent available studies supporting the use of EUS-FNA/FNB for patients with PDAC come from researchers at the Cold Spring Harbor Laboratory, Harbor, NY, USA. In the prospective clinical trial, they showed a success rate of isolation and propagation of PDAC organoids from EUS-FNA/FNB specimens of ~70–80% (46, 47). Interestingly, they proved that two FNB needle passes were not superior as compared to a single one, even though this result could be attributed to a bias due to the learning curve effect in organoid creation (47).

Based on the above evidence, the surgical approach for PDO creation may still be considered the most comprehensive methodology for an appropriate tumor biology assessment, thanks to a large amount of tissue available and to the preservation of the entire tumor environment. On the counterpart, the endoscopic approach is gaining increasing relevance in the better understanding of tumor behavior of malignancies not susceptible to surgical resection. However, further studies and technical improvements are still needed in order to ameliorate tissue retrieval and analysis and to support the EUS approach as a valuable alternative to the surgical technique (40).

## PC PDO Platforms are an Essential Tool for Personalized Therapy

Most cancers are heterogeneous diseases. Several types of cancer with the most disparate genomic and molecular features can affect the same organ. Moreover, lesions can present several cancer clones, each with specific characteristics and differential levels of sensitivity to treatment. Indeed, heterogeneity provides the fuel for drug resistance and is one of the main reasons for the lack of response to therapeutic interventions. Therefore, taking into account, tumor heterogeneity is essential for the development of effective therapies.

The capability of PDOs to represent a bona fide copy of *in vivo* cancers makes them ideal models for PC drugs screening and *ex vivo* pharmacogenomic profiling.

Driehuis et al. (48) established a platform of 27 PDO models from PC that were used for a high-throughput drug screening with a panel of 76 different therapeutic agents. They observed that organoids derived from different patients had different responses to various drugs, highlighting the problem of response heterogeneity in PC. They found that PDOs harboring *MAP3K1* and *PIK3R1* copy number alterations showed particular sensitivity to lapatinib a HER2/EGFR inhibitor. Moreover, PDOs with a deletion of the *MTAP* gene were more sensitive to the selective protein arginine methyltransferase 5 (PRMT5) inhibitor EZP01556. The *MTAP* gene is located on chromosome 9p21 near CDKNA2, and it is co-deleted with this gene in 90% of the samples. The PRMT5 inhibition is synthetic lethal for cells with loss of *MTAP* (49). At least one copy of *MTAP* was found in a subset of PDOs that were sensitive to EZP01556.

Another study characterized the genome and transcriptome profile of a library of 66 PDO cultures derived from primary PDAC and developed a specific drug-testing pipeline demonstrating a quite similar sensitivity profile between PDO response and outcome of the patient (50). In detail, the authors tested five standard-of-care chemotherapeutics and 21 investigational agents. About 33% of analyzed PDO models were resistant to all chemotherapeutic agents, and 21 of them were tested for alternative targeted agents finding extreme sensitivity in half cases. In addition, a sequential PDO model derived from the same patients predicted *in vitro* the acquisition of chemotherapy resistance according to the time point of clinical progression. With this innovative approach, drug sensitivity information can be obtained for each PDO model within a clinically meaningful timeframe. The incorporation of PDO pharmacotyping in clinical practice will greatly enhance precision medicine and finally patient outcome. Of note, non-negative matrix factorization (NMF) clustering classification of PDO transcriptomes data indicated the existence of two different stable clusters, one enriched for TGF-β signaling and EMT, prevalently concordant with Basal-like PDO classification, and another linked to xenobiotic metabolism, fatty acid metabolism, and oxidative phosphorylation, which fell in the classical subtype (50). A recent study also pharmacotyped 29 PDO cultures from resected patients with PC to test chemotherapy sensibility showing the feasibility of this strategy within a clinically meaningful timeframe for adjuvant therapy decision (51). Indeed, the clinical translation of PDO-driven decision-making is straightly linked to the ability to shorten the time from tissue sampling to sensibility test results. As metabolism changes precede cell viability reduction, it has been proposed the use of optical metabolic imaging to fasten drug response measurement (52). Through this high-resolution fluorescence microscopy technique, the authors correctly classified as non-responders, three patients who displayed recurrence within 1 year, and as responders, four patients who remained free of recurrence in the same time frame.

Altogether, these studies clearly suggest that pancreatic PDO may serve as biobanks for novel drug discovery or as a rapid screening individualized tool for personalized medicine (53). Several clinical trials employing PC PDO are currently ongoing (Table 1).

**Table 1:** Clinical Trials employing pancreatic cancer PDO.

**Study Id**	**Registry**	**Study Design Classification**	**Disease Characteristics**	**Estimated Enrolment**	**Status**	**Official Title**	**Study Start Date**	**Estimated Study Completion Date**	**Organoids Establish** **ment**	**Correlation with treatment**	**PDO related outcome**
NCT03140592	US NLM-NHI	Observational Model: Cohort -Prospective	Pancreatic cancer, pancreatic cyst	300 participants	Recruiting	EUS-guided Biopsy of Pancreatic Mass Lesions for Developing Patient-Derived Cancer Models	January, 2015	January, 2023	Yes	No	Successful generation of pancreatic organoids
NCT03990675	US NLM-NHI	Intervention Model: Single Group Assignment	Pancreatic cancer	50 participants	Recruiting	Evaluation and Comparison of the Growth Rate of Pancreatic Cancer Patient-derived Organoids Generated From Matched Fine Needle Aspirations (FNA) and Fine Needle Biopsies (FNB)	December, 2018	August, 2021	Yes	No	Growth rate of pancreatic organoids
NCT03500068	US NLM-NHI	Intervention Model: Single Group Assignment	Locally advanced pancreatic cancer or metastatic pancreatic cancer	30 participants	Recruiting	Establishing Organoids From Metastatic Pancreatic Cancer Patients, the OPT-I Study	September, 2017	September, 2022	Yes	Yes	Developing organoids from advanced pancreatic cancer patients that predict non response or response; Secondary outcome: Functional studies will be done with the patient derived organoids to find biomarkers that correlate with response in organoids and patients
NCT04469556	US NLM-NHI	Intervention Model: Parallel Assignment	Advanced or metastatic pancreatic cancer	150 participants	Recruiting	Pancreatic Adenocarcinoma Signature Stratification for Treatment	October, 2020	September, 2023	Yes	Yes	Concordance between organoid transcriptomic profiles (RNAseq) and patient transcriptomic profiles, Concordance between chemotherapy sensitivity signature predictions and response to first line treatment
NCT03544255	US NLM-NHI	Observational Model: Cohort	Pancreatic cancer	50 participants	Recruiting	Drug Screening of Pancreatic Cancer Organoids Developed From EUS-FNA Guided Biopsy Tissues	May, 2018	June, 2021	Yes	No	Number of organoids successfully generated from pancreatic cancer biopsies; Secondary outcome: Response of the pancreatic cancer organoids to the selected anti-cancer drug
NCT02869802	US NLM-NHI	Observational Model: Cohort -Prospective	Pancreatic cancer	190 participants	Recruiting	Prospectively Defining Metastatic Pancreatic Ductal Adenocarcinoma Subtypes by Comprehensive Genomic Analysis	October, 2016	September, 2023	Yes	Yes	Participants will undergo a tumour biopsy at baseline and an optional tumour biopsy at disease progression. Success rate of establishing patient-derived organoids. Engraftment rate of patient-derived xenografts
NCT02436564	US NLM-NHI	Observational Model: Case-Only -Prospective	Cholangiocarcinoma, hepatocellular carcinoma, pancreatic cancer	75 participants	Unknown	A Study Designed to Develop *in vitro* Models of Liver, Biliary and Pancreatic Cancer for the Investigation of Tumour Biology and Potential Therapies	July, 2015	NA	Yes	Yes	Generation of organoids, genomic DNA and RNA sequencing, application of genome editing technology, investigation of anti-tumour drugs, investigation of biological and functional properties of tumour cells, transplantation of organoid lines into animal models.
ChiCTR1900 028000	ChiCTR	Observational	Pancreatic cancer	50 participants	Unknown	Tumor evolution and drug response in pancreatic cancer organoids by means of EUS-guided fine-needle aspiration	December, 2019	December, 2021	Yes	Yes	Evaluation the feasibility and safety of EUS-FNA sampling in pancreatic cancer patients to produce human pancreatic cancer organoids on the basis of preliminary research and experiments. Comparison of organoid sensitivity to different chemotherapy with clinical patient response.
ChiCTR2000 037214	ChiCTR	Observational	Pancreatic cancer	200 participants	Recruiting	Research on individualized treatment of pancreatic cancer patients based on organoid model	August, 2020	September, 2023	Yes	Yes	Generation of a living bank of organoids and matched bioinformation database, characterization of organoids sensitivity to a large-scale drug candidates.
ChiCTR-OOC-17012057	ChiCTR	Observational	Pancreatic cancer	50 participants	Unknown	3D Pancreatic cancer organoid models developed from EUS-FNA guided biopsy tissues for drug screening as a prospective study	July, 2017	September, 2019	Yes	Yes	Set up organoid models for pancreatic cancer patients, genotyping of organoids and analyses of the molecular mechanisms for the drug resistance.
CTRI/2017/05/ 008512	CTRI	Observational, Prospective Pilot study	Metastatic pancreatic cancer	20 participants	Unknown	To assess the correlation chemotherapy effect on the tumor and in the patient derived organ like creation from pancreatic cancer cells. Do patient derived organoids predict therapeutic response in pancreatic cancer patients with advanced disease?	May, 2017	NA	Yes	Yes	Comparison of the responses to chemotherapy drugs in metastatic pancreatic cancer patients and *in vitro* with patient derived organoids. Identification markers for drug response with the help of the patient derived organoid.
DRKS000 21088	DRKS	Observational	Pancreatic cancer	118 participants	Recruiting	Generation of PDAC organoides by means of EUS-guided fine needle biopsy sampling for personalized cancer treatment	April, 2019	NA	Yes	No	Organoid generation by co-culture mimicking microenvironment
TCTR20200 827007	TCTR	Interventional	Pancreatic cancer	28 participants	Recruiting	Successful rate of creation pancreatic cancer organoids by EUS-guided fine needle biopsy sampling	August, 2020	October, 2021	Yes	No	Successful generation of organoid culture by EUS-FNB sampling

*US NLM-NHI, United States National Library of Medicine-National Institutes of Health; ChiCTR, Chinese Clinical Trial Registry; TCTR, Thai Clinical Trials Registry; CTRI, Clinical Trials Registry India; DRKS, Deutsches Register Klinischer Studien*.

## PDO Modeling to Study Pancreatic Precursor Lesions

Patient-derived-organoids proved to be instrumental models to study PC development. In fact, two studies already showed that it is possible to establish organoids from IPMN, a key precursor lesion of PDAC. Beato et al. (54) established a biobank of IPMN PDOs and performed histological and molecular analyses to show that PDO preserved the parental features. The expression levels of the IPMN markers MUC5AC and CK19 were consistent in the parental tumor and organoid in most of the cases. Whole exome sequencing (WES) also showed that IPMN PDOs retain the same genomic features. Similarly, Huang et al. (55) generated PDOs from normal pancreatic ducts and IPMNs and performed both WES and RNA-sequencing. Interestingly, they found few genes differentially expressed between normal pancreatic duct and IPMN organoid samples. The most significantly downregulated gene was the transcription factor *FOXA1*. Validation analysis performed on a large cohort of IPMNs, PDAC, and normal ductal samples showed a substantial downregulation of *FOXA1* in both IPMNs and PDAC demonstrating that the loss of this transcription factor is a key early event in PDAC oncogenesis.

A cutting-edge tool in PDO modeling is the organoid-on-a-chip technology that guarantees high-throughput screening tests with optimized and standardized culture conditions reducing potential differences in organoid size, shape, and geometry. The integration of this organoid-on-a-chip with microfluidic technology using continuously perfused chambers further improved the biomimicry of the model reproducing the microvascular system of large-scale tumors (56).

Recently, it has been demonstrated that organoid-on-a-chip technology could be used to establish patient-specific pancreatic duct models (57). The authors performed single-cell RNA sequencing analysis to study the differentiation of pancreatic duct-like organoids from human-induced pluripotent stem cells grown on a microwell chip. More importantly, they highlighted the potential of this study model to investigate ductal markers relevant to pancreatic carcinogenesis. Together with prognostic and early-stage PC biomarkers discovery, this microwell chip can allow the exploration of pancreatic ducts and stromal cells crosstalk. Indeed, the authors used a microwell chip with fluidic-independent hexagonal arrays to create a cross-contamination-free co-culture system of organoids of patients and pancreatic stellate cells. These stomal cells can switch from a quiescent to an active state under specific stimuli-like inflammation and tumor development allowing the study of PC pathogenesis (57).

## PDOS and Radiotherapy

Some studies also showed how PDO platforms might be utilized to study the radiotherapy response and find new ways to achieve radiosensitivity. Nicosia et al. (58) utilized a platform of PDAC PDOs to study the combinatorial effect of magnetic field (MF) and radiotherapy. They found that PDOs displayed heterogeneous responses to irradiation, where few were particularly sensitive to radiotherapy. They treated PDOs with a dose of 1.5 T MF integrated into the MR-linac, with an exposed time 60-min long, demonstrating that MF adds therapeutic effects when administered before X-rays in all the PDOs tested, whereas MF alone displayed no biological effect. The reduced cell viability of PDOs subjected to combined MF and radiotherapy was associated with a reduced organoid size and increased apoptosis, whereas the biological mechanisms are still unknown.

## PC PDOS as a Tool to Study Stroma/Tumor Cell Interaction

On average, about 70% of the PC tumor bulk is constituted of stroma that influences disease pathogenesis and response to therapy (9). Single-cell analysis revealed extensive stromal heterogeneity across tumors with the existence of functional opposite stromal cells (13).

Since conventional stereotypical Matrigel-embedded organoids contain only epithelial cells, any investigation of the TME in this system can be challenging and necessitates the addition of exogenous stromal cell types to fully probe cell-cell interplay and faithfully reproduce real *in vivo* tumor–niche composition and its cellular diversity. Indeed, stroma cells directly or indirectly interact with cancer cells to modify disease presentations influencing tumor behavior and immune–tumor interaction. Thus, miscellaneous reconstitution approaches have been proposed for modeling disease ecosystems trying to reproduce the symbiotic interactions seen in the whole organism. For instance, such co-culture strategies have been utilized to supplement PDOs with CAFs, a tumor-specific stromal element of TME that is thought to exert a pleiotropic role in cancer development (35, 59). Seino et al. (59) demonstrated the existence of different degrees of Wnt-niche dependencies among human PDACs. In detail, they generated a library of 39 confirmed patient-derived PDAC organoids and identified among them three functional subtypes, a Wnt-producing subtype, a Wnt-non-producing subtype that required exogenous Wnt supply from adjacent CAFs, and an R-spondin-independent subtype capable of growing without both Wnt and R-spondin. Moreover, the CAF-conditioned medium failed to promote organoid proliferation and co-culture without physical contact indicating that direct physical juxtacrine interaction between PDAC organoids and CAFs was required to efficiently support tumor growth through pro-tumorigenic niche signal and Wnt production. Interestingly, when subcutaneously injected in mice, Wnt-non-producing organoids did not generate tumor mass, whereas co-transplantation with CAFs increased engraftment rate success. A similar co-culture model using green fluorescent protein (GFP)-labeled tumor-derived murine pancreatic organoids plus mCherry-labeled murine pancreatic stellate cells (PSCs) revealed cooperative interactions between CAFs and PDO organoids recapitulating the *in vivo* CAF heterogeneity. In this model, under co-culture conditions, PSCs grew in tight contact with cancer cells, became activated, and acquired CAF phenotype with ECM deposition, thus reproducing *in vitro* the PDAC desmoplastic reaction. Notably, PSCs differentiated in two CAF subpopulations, preferentially standing in distinct locations respect to cancer organoids: α-smooth muscle actin (αSMA)^high^ IL-6^low^ myofibroblastic CAFs (myCAFs) directly interacting with cancer cells and peripheral αSMA^low^ IL-6^high^ inflammatory CAFs (iCAFs) scattered in areas distant from PDAC cells able to promote tumorigenesis through inflammatory cytokine production. This organization in organoid/CAF co-cultures reflects PDAC myCAF and iCAF spatial distribution pattern in human tissue. Finally, through modification of co-culture condition, authors demonstrated that CAF subtypes coexist in tumor as two dynamic and reversible phenotypes based on their location and biochemical niche within tumor microenvironment (60). In addition, another study (61) showed that, in symbiotic co-culture systems of organoids and CAFs, diversification of CAFs depends on tumor-derived stimuli they are exposed to. Indeed, tumor cells secrete specific mediators to guide CAF differentiation in myCAF or iCAF, thus sustaining CAF heterogeneity. They demonstrated that iCAF formation is induced by tumor-secreted IL-1 that activates a LIF/JAK/STAT-dependent cytokine cascade, whereas tumor-secreted TGF-β antagonizes JAK/STAT signaling by IL-1R1 downregulation and promotes differentiation into myCAF. Additionally, they identified a third minor subtype of CAFs with αSMA/p-STAT3 double positivity, which could be an intermediate cell state, confirming the phenotype plasticity of CAFs.

Moreover, it has been proposed an air-liquid interface (ALI) PDO method to obtain and propagate primary clinical tumors preserving the multicomponent feature of TME with tumor parenchyma and stromal elements, including functional tumor-specific infiltrating lymphocytes (62). This technique guarantees to retain and expand within organoids the endogenous stroma and immune cells present in the tumor tissue. In particular, a large-scale study on 100 resected tumor samples including PDAC demonstrated that ALI PDO cultures highly preserve integrated stromal CAFs and tumor architecture (63).

Microenvironment heterogeneity is an emergent field of study in cancer that is still in its infancy. Interestingly, this heterogeneity is evident even in precursor lesions and during multistep progression from non-invasive IPMNs to PDAC in a stage-specific manner (64).

In the next future, more stroma subpopulations will likely be identified, and PDO-based strategies might become the preferential discovery tool.

Altogether, these studies highlighted the potential applications of new organoid-centered assays, i.e., organoids/stroma co-culture systems, in drug development to find new putative targets or pathways to specifically hit stroma–PC crosstalk.

## PC PDOS as Immuno-Oncology Models

A further complexity step in the PDO research development is the establishment of a hybrid culture of organoids with immune cells that enable *ex vivo* modeling of complete PC microenvironment and immune–tumor interactions.

This can be achieved by the secondary addition of autologous/heterologous peripheral immune cells separately expanded to organoids or by technique to co-expand endogenous tumor infiltrating immune cells with tumor cells.

Tsai et al. (35) developed and characterized a platform of patient-matched organotypic models incorporating human PC organoids together with CAFs and T cells from the same patients. The PDOs were directly established from ascitic fluid samples, resected primary and metastatic tumors, and unique rapid autopsy specimens were taken within 1–2 h from death. The authors showed that patient-matched peripheral blood lymphocytes, growing in the liquid phase above Matrigel dome, are able to infiltrate the Matrigel migrating toward organoids. Such 3D *in vitro* disease reproduction strategies, which are representative of the major components of the tumor microenvironment, are suitable for the study of tumor-stroma interplay and the assessment of immunotherapy drugs such as checkpoint inhibitors (35).

As one of the major features of PC is the highly immunosuppressive microenvironment with the activation of multiple redundant mechanisms to evade immune surveillance, additional preclinical models, incorporating other immune cells besides lymphocyte, could respond to the increased demand for a reliable predictive platform for PDAC therapies management.

The general failure of anti-PD-1 targeting in PC may be due to numerous suppressive immune cell types accumulating in early PanIN lesions and persisting through cancer progression (65). Within the immunosuppressive PDAC milieu, MDSCs play a key role in PD-1/PD-L1 checkpoint activation and are able to hamper cytotoxic T-cell activity through sequestration of L-arginine and L-cysteine and production of reactive oxygen species (66, 67).

A recent study established co-cultures of PDO and matched polymorphonuclear (PMN)-MDSCs in order to test the efficacy of combinatorial therapies involving PD-1 inhibition and MDSC depletion strategy. As expected, PD-L1-expressing organoids were refractory to nivolumab treatment in the presence of PMN-MDSCs, whereas the depletion of arginase 1-positive PMN-MDSCs by cabozantinib reverted resistance, maximizing the effect of anti-PD-1/PD-L1 treatment (68).

These disease-relevant models may be valuable to study cross-talk between leukocytes and organoids, cytotoxicity of engineered lymphocytes, clonal expansion of personalized anti-tumor T cells, and the evaluation of immunotherapy effects such as checkpoint inhibitors.

## Conclusions

Malignancies heterogeneity is a crucial concern in cancer treatment management, as it often triggers resistance mechanisms and tumor relapse. Marked tumor heterogeneity among different tumors and even within each tumor is a key mark of PC. PDOs from PC specimens fully recapitulate tumor heterogeneity in phenotype and cytoarchitecture while retaining molecular characteristics of primary cancer they originate from.

The PDO technology is having a growing impact on the translational cancer research field. Tumor organoids can be easily expanded, cryopreserved, genetically edited, and co-cultured with immune cells, CAFs, or other cell components becoming an even more biologically relevant clinical avatar.

The PDO potentiality as a personalized medicine platform is currently under investigation, and the discovery of their power is still in its infancy. Research efforts are now globally pointing to include tumor PDOs in clinical trials for drug screening and repurposing strategy.

## Author Contributions

GP, CC, and AL: conceptualization. GP and AA: formal analysis and resources. GP, AA, AL, GQ, CC, AE, GR, and FA: writing—original draft preparation. SA, GC, and GT: writing—critical review and editing. GT: supervision. All authors have read and agreed to the published version of the manuscript.

## Funding

This work was supported by the AIRC IG (grant no: 26330), Institutional Funds of University Cattolica Del Sacro Cuore UCSC project D1 (grant no: 2019–2021) to GT, and My First AIRC Grant Luigi Bonatti e Anna Maria Bonatti Rocca, (grant no: 23681) to CC.

## Conflict of Interest

The authors declare that the research was conducted in the absence of any commercial or financial relationships that could be construed as a potential conflict of interest.

## Publisher's Note

All claims expressed in this article are solely those of the authors and do not necessarily represent those of their affiliated organizations, or those of the publisher, the editors and the reviewers. Any product that may be evaluated in this article, or claim that may be made by its manufacturer, is not guaranteed or endorsed by the publisher.
